# Soil evaporation and plant water use strategy of riparian forests in extremely arid deserts, northwestern China: a stable isotope perspective

**DOI:** 10.3389/fpls.2026.1797109

**Published:** 2026-04-10

**Authors:** Qi Zhang, Qi Feng, Yonghong Su, Wei Liu, Meng Zhu, Yuanyuan Xue

**Affiliations:** 1 Key Laboratory of Ecological Safety and Sustainable Development in Arid Lands, Northwest Institute of Eco-Environment and Resources, Chinese Academy of Sciences, Lanzhou, China; 2 University of Chinese Academy of Sciences, Beijing, China

**Keywords:** evaporation loss, extremely arid deserts, riparian forests, stable isotopes, wateruse strategy

## Abstract

**Introduction:**

In extreme arid regions, *Populus euphratica* (*P. euphratica*) and *Tamarix ramosissima* (*T. ramosissima*) play vital ecological and landscape roles, but their survival and regeneration are severely limited by water scarcity. Since soil evaporation and plant transpiration represent the major pathways of water loss in these ecosystems, quantifying soil evaporation rates and clarifying plant water-use strategies are crucial for supporting vegetative growth and maintaining population stability.

**Methods:**

This study investigated water movement in *P. euphratica*, *T. ramosissima*, and their mixed stand using multi-source isotope data collected from June to September.

**Results:**

Soil evaporation exhibited consistent patterns across all stands, being most pronounced in the shallow layer (0–40 cm) and declining exponentially with depth, while the deep soil layer (>200 cm) was primarily recharged by groundwater. Although vegetation type introduced some variability in evaporation rates, these differences did not reach statistical significance. Both species primarily extracted water from the 100–300 cm soil layer and groundwater, with *P. euphratica* relying more heavily on soil water at 100–200 cm depth and *T. ramosissima* utilizing more groundwater, reflecting its greater drought tolerance. In mixed stands, this divergence in water use intensified interspecific competition, resulting in lower soil moisture and a more rapid decline in groundwater levels compared to pure stands, a phenomenon linked to differences in root distribution.

**Discussion:**

To maintain the structure of desert riparian forests, ecological water conveyance must be carefully managed to sustain groundwater levels within a suitable range for vegetation. Such management would help prevent *T. ramosissima* dominance and facilitate the survival, regeneration, and sustainable development of *P. euphratica*.

## 1 Introduction

As essential components of azonal vegetation in arid landscapes, riparian forests preserve regional ecological security and landscape integrity by delivering important ecosystem services (Ling et al., 2019, 2020; Macfarlane et al., 2017). With well-developed root systems, these vegetation belts enhance soil resistance, stabilize riverbanks, and act as frontline barriers against desertification (Ding et al., 2017; Huang et al., 2016; Zhao et al., 2023). However, under escalating climate change, arid regions are experiencing more frequent extreme events and altered precipitation patterns (Chen et al., 2022; Luo et al., 2021; Fraser et al., 2011; Muñoz-Villers et al., 2018; Wang et al., 2022), leading to declining water availability (Huang et al., 2017a; Schlaepfer et al., 2017; Tietjen et al., 2010). These changes amplify degradation risks in desert riparian forests, fragile habitats already stressed by drought and salinity (Huang et al., 2017b; Li et al., 2024; Prăvălie, 2016). Ecosystem sustainability thus depends on understanding water consumption processes dominated by soil evaporation and plant transpiration (Dubbert et al., 2014; Sun et al., 2022; Yuan et al., 2015). Accurately quantifying soil evaporation loss and elucidating plant water-use strategies across physiological stages are therefore crucial for improving water-use efficiency and minimizing unproductive losses.

Stable hydrogen and oxygen isotopes (δ²H and δ^18^O) provide a powerful tool for investigating these processes, serving as intrinsic tracers that track water movement from precipitation through soil to plant uptake and atmospheric diffusion (Bowen et al., 2019; Cao et al., 2025; Huang et al., 2019; Penna et al., 2018). Along this pathway, soil evaporation preferentially releases lighter isotopes, enriching the remaining water, a process especially critical in arid regions where evaporation accounts for substantial water loss (Han et al., 2019; Quade et al., 2018; Zhang et al., 2024; Zhou et al., 2020). The line-conditioned excess (lc-excess) qualitatively indicates the extent of evaporation fractionation (Benettin et al., 2018; Gibson et al., 2008; Landwehr and Coplen, 2006; Sprenger et al., 2017), while the Craig-Gordon model enables quantitative estimation of evaporation loss rates (Sprenger et al., 2017; Yong et al., 2020; Zhai et al., 2019). After infiltration, soil water moves mainly through preferential flow, with isotopes reflecting mixing and residence times across layers (Dai et al., 2022; Kleine et al., 2020; Tan et al., 2017; Zhu et al., 2022). Root water uptake at the soil-plant interface represents another critical component of regional water cycling (Geris et al., 2017; Hardanto et al., 2017; Wang et al., 2017), with xylem water isotopes revealing selective absorption patterns influenced by soil moisture distribution (Alam et al., 2018; Liu et al., 2023; Rothfuss and Javaux, 2017; Wang et al., 2021). Under global climate change, altered precipitation and intensified droughts are reshaping arid-region ecohydrology and challenging plant growth (Zhao and Wang, 2018). In shared habitats, species often exhibit divergent water-use strategies to adapt to drought and competition (Allen et al., 2019; Huo et al., 2018; Song et al., 2020; Wang et al., 2017). Given the fragile ecosystems and water scarcity in arid regions (Schlaepfer et al., 2017; Zhao and Wang, 2018), analyzing soil-plant water dynamics using these isotopic tools is essential for informing vegetation management and promoting sustainable ecosystem development.

In the extreme arid environments of Northwest China, these isotopic approaches are particularly valuable for investigating the water use dynamics of foundation species that sustain fragile desert ecosystems. Among these species, *Populus euphratica* and *Tamarix ramosissima* are keystone species in desert riparian ecosystems, valued for their adaptability to salinity, drought, and fluctuating groundwater levels (Cao et al., 2011; Sun et al., 2022). Through deep root systems that extract and transpire groundwater, these plants maintain surface-groundwater hydraulic connectivity, stabilize dunes, and mitigate salinization (Chen et al., 2016; Li et al., 2019a). Their distinct phenology and morphology confer aesthetic value, making them characteristic landscape units in arid regions (Miao et al., 2020; Su et al., 2020; Sun et al., 2022). The Heihe River Basin, a representative inland basin in arid northwest China, is a key focus of global dryland ecohydrology research (Cheng et al., 2014; Li et al., 2018). Its downstream desert-oasis ecosystem helps restrain Badain Jaran Desert expansion, largely sustained by the sand-fixing capacity of *P. euphratica* and *T. ramosissima* communities (Cao et al., 2011; Sun et al., 2022). Hydrologically, plant water uptake here depends on groundwater recharged by river runoff, intermittent flood infiltration, and limited precipitation, exhibiting distinct spatiotemporal patterns (Liu et al., 2015; Sun et al., 2022; Yu et al., 2018). Investigating water transport in these foundation communities is therefore crucial for understanding co-evolutionary mechanisms across the groundwater-soil-plant-atmosphere continuum and for identifying ecological restoration thresholds in arid inland basins.

Despite previous research on water use in arid regions (Wan et al., 2022; Wu et al., 2016; Ye et al., 2019), key uncertainties remain concerning these keystone species and their environment. First, while soil evaporation is known to be a major water loss pathway in drylands, its precise contribution to the overall water balance in desert riparian forests remains poorly quantified. Second, although *P. euphratica* and *T. ramosissima* are acknowledged as keystone species, their water-use strategies across different forest types (pure and mixed stands) and their potential differentiation when coexisting in the same habitat have not been systematically compared. Third, the hydrological connectivity and interactions among precipitation, soil water, groundwater, and plant xylem water in the groundwater-soil-plant-atmosphere continuum of this extreme arid region have yet to be comprehensively characterized, limiting our ability to predict ecosystem responses to ongoing climate change. To address these uncertainties, this study focused on *P. euphratica*, *T. ramosissima*, and their mixed forests in the lower Heihe River Basin. Using stable isotope data from precipitation, soil water, stem water, and groundwater collected from June to September 2024, the research aims to systematically addressed three primary objectives: (1) characterize the isotopic composition of different water sources and examine interactions among hydrological components; (2) analyze soil water dynamics, emphasizing evaporation-mediated transport processes and their environmental drivers; (3) quantify the proportional contributions of potential water sources and elucidate water-use strategies of *P. euphratica* and *T. ramosissima* in pure and mixed stands, to reveal how coexisting species partition limited water resources. The findings are expected to reveal central water loss mechanisms in the desert-oasis water cycle, thereby providing a theoretical foundation for improved water resource management and ecological restoration in degraded oasis ecosystems.

## 2 Materials and methods

### 2.1 Study area

The Ejin Oasis (40°57′–42°33′ N, 100°9′–101°46′ E) is located on the alluvial plain of the lower Heihe River in Alxa League, Inner Mongolia Autonomous Region, China. As a typical desert oasis within an extreme arid zone, it is bordered by the Badain Jaran Desert to the north (Shi et al., 2024). The region is influenced by both the Siberian High and the thermal dynamics of the Tibetan Plateau, resulting in a temperate continental extreme arid climate. Mean annual precipitation is only 35.5 mm, with approximately 70% occurring as short-duration torrential rainfall from July to September. In contrast, the annual potential evaporation reaches 3152.5 mm, yielding a dryness index of 80–100, which far exceeds the threshold for arid regions (Li et al., 2025). The mean annual temperature is 8.3 °C, with recorded extremes of 43.1 °C and –37.6 °C; diurnal temperature variations often exceed 20 °C (Mao et al., 2024). The area experiences 15 to 20 sand-dust storm days per year, predominantly from March to May, including 3–5 severe sandstorms annually (Peng et al., 2020). Soils are predominantly poor and saline alluvial types, with low organic matter content and high moisture evaporation rates, frequently leading to the formation of surface salt crusts (Fan et al., 2018). *P. euphratica* and *T. ramosissima* form the core plant species that sustain the desert oasis ecosystem, maintaining its fragile ecological balance through high tolerance to drought and saline-alkali stress (Shiyomi et al., 2024). By the late 20th century, the cessation of flow in the Heihe River had triggered severe vegetation degradation driven by soil salinization and desertification. Although ecological water conveyance projects initiated after 2000 have promoted partial recovery of riparian vegetation, the periphery of the oasis continues to face persistent threats from wind erosion and groundwater over-extraction (Fan et al., 2018).

### 2.2 Sample collection

**Figure 1:**
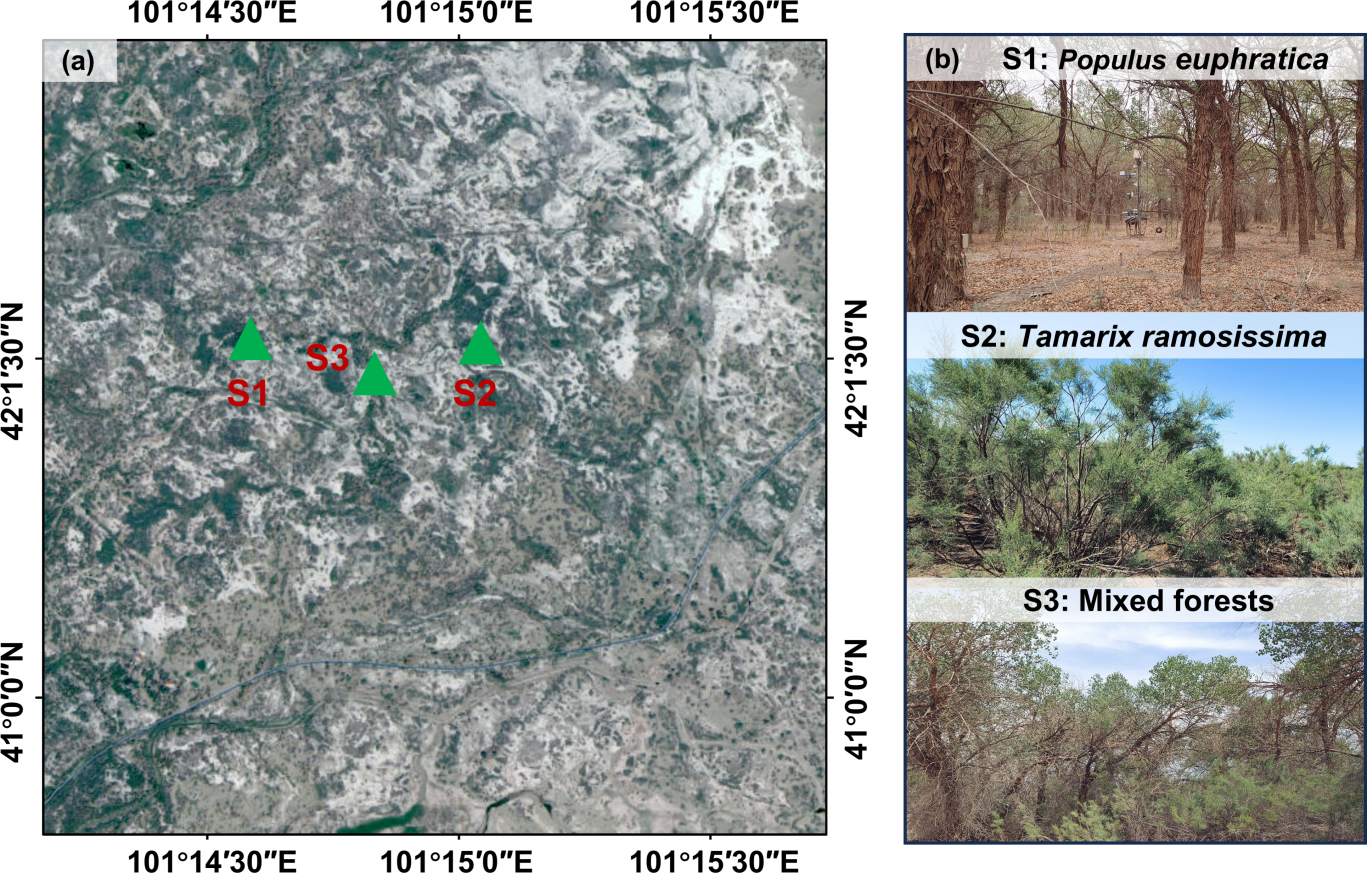
Overview of the study area **(A)** sampling site, and **(B)** photographs of S1 (*Populus euphratica*), S2 (*Tamarix ramosissima*) and S3 (Mixed forests). The remaining figures, tables, and their captions are correct, and all figures are of the highest quality/resolution.

Based on the research objectives and the spatial distribution characteristics of vegetation communities in the Ejin Oasis of the lower Heihe River (**Figure 1**), three representative 20 m × 20 m vegetation plots were established in the region. Specifically, S1 comprised of *P. euphratica*, S2 consisted of *T. ramosissima*, and S3 was a mixed forest (**Table 1**). The stands were spaced within 1 km of each other to maintain relative consistency in regional hydrothermal conditions and topographic factors, thereby reducing potential confounding effects due to spatial heterogeneity. Within each stand, three randomly located 5 m × 5 m sampling points were established. Monthly sampling was conducted throughout the plant growing season (June to September) in 2024, collecting samples from precipitation, stems of dominant plant species, soil profiles (0–300 cm depth), and groundwater.

**Table 1:** Plant morphometric parameters in stands.

Growth metrics	S1 (*P. euphratica*)	S2 (*T. ramosissima*)	S3 (*P. euphratica*)	S3 (*T. ramosissima*)
Height (m)	6.03	2.90	6.61	2.44
Diameter at Breast height (DBH, cm)	22.03	2.72	23.04	2.28
Crown Width (m)	4.71×4.27	2.46×2.16	4.24×4.11	2.17×1.94

Given the small size of individual rainfall events and the high spatiotemporal heterogeneity of precipitation within the arid oasis, direct real-time rainfall collection inside the vegetation plots was not feasible. Accordingly, precipitation samples were collected using standardized rainfall collection devices installed at the nearby Alxa Eco-Hydrological Station. After each rainfall event, water from the rain gauge was transferred into 20 mL polyethylene sampling bottles, with three replicates collected for every rainfall event. The bottles were immediately sealed with three layers of Parafilm to minimize isotopic fractionation and were then stored at –4 °C until δ²H and δ^18^O analysis could be performed.

During the plant growing season, soil samples were collected monthly in the early to mid-month across all study plots. At each sampling points, three randomly selected sampling points were excavated to a depth of 300 cm to enable vertically stratified sampling. Within the 0–100 cm layer, samples were taken at 10 cm intervals, and from 100–300 cm at 20 cm intervals, with three replicates collected at each depth while deliberately avoiding primary root zones. To more precisely characterize plant water uptake zones, the soil profile was divided into four depth classes based on oxygen isotope composition and soil water content: 0–40 cm (surface layer), 40–100 cm (subsurface layer), 100–200 cm (intermediate-depth layer), and 200–300 cm (deep layer). This stratification captures the vertical gradient of evaporative enrichment and facilitates comparison of plant water uptake patterns across soil layers with distinct hydrological characteristics. Upon collection, each soil sample was divided into two portions. One portion was placed into a pre-weighed aluminum box for immediate wet weight measurement before oven-drying at 105 °C for 24 hours to determine gravimetric water content based on mass difference. The other portion was promptly transferred to an 80 mL polyethylene bottle, sealed with Parafilm to prevent atmospheric exchange, and cryopreserved at –20 °C. Groundwater samples were also collected monthly at corresponding profile locations, as the water table remained stable at approximately 3 m depth. After allowing groundwater seeping from the profile to accumulate steadily for 5 minutes, samples were collected using 20 mL polyethylene bottles. These were immediately sealed with Parafilm and stored at 4 °C until isotopic analysis.

Plant xylem sampling was conducted simultaneously with soil collection during clear morning hours (08:00 to 11:00), a period chosen for its stable transpiration rates and equilibrium in xylem water transport. Within each plot, five conspecific individuals with uniform morphometric traits (**Table 1**) were selected. Sun-exposed mature branches measuring 0.3–0.5 cm in diameter were excised. After removing the phloem, the xylem tissues were cut into 3–5 cm segments. These segments were immediately placed into 20 mL amber glass vials, with five segments placed in each vial to ensure sufficient sample mass for water extraction. The vials were then sealed with Parafilm and stored frozen at –20 °C.

### 2.3 Sample measurement and analysis

#### 2.3.1 Experimental analysis

Water extraction from soil and plant samples was performed using cryogenic vacuum distillation. Sample masses were accurately measured before and after extraction to confirm extraction efficiency of ≥98%. After natural thawing to ambient temperature, the extracted water was filtered through 0.45 μm membrane syringe filters under strict single-use protocols to prevent cross-contamination. Aliquots (0.5–1.5 mL) were transferred into 2 mL vials, which were immediately sealed with septa and stored at –4 °C until isotopic analysis. The δ²H and δ^18^O compositions were determined using a Los Gatos Research (LGR) Liquid Water Isotope Analyzer, with analytical precisions of ±0.5‰ and ±0.1‰, respectively (Equation 1). Each sample received six replicate injections, with the first two discarded to mitigate isotope memory effects and the mean of the remaining four used to determine the final isotopic values. To address known spectral contamination issues in xylem water analyses, correction algorithms provided by LGR were applied to the raw data.

(1) $$\begin{array}{c} \delta \left(‰\right)=\left(\frac{R_{sample}-R_{standard}}{R_{standard}}\right)\times 1000 \end{array}$$

where R_sample_ denotes the ratio of heavy to light isotope in the sample, and R_standard_ is the corresponding ratio in the international reference material (Vienna Standard Mean Ocean Water, VSMOW).

#### 2.3.2 Lc-excess

The line-conditioned excess (lc-excess) is a parameter that quantifies the deviation of δ²H and δ^18^O composition from the Local Meteoric Water Line (LMWL). It is commonly used to qualitatively indicate non-equilibrium kinetic fractionation resulting from evaporation (Equation 2). Due to evaporative processes, soil water, river water, and groundwater typically exhibit negative lc-excess values, where more negative values correspond to stronger evaporation intensity. Variations in lc-excess in local atmospheric precipitation are generally influenced by different moisture sources, although its annual average remains zero.

(2) $$\begin{array}{c} lc-excess=\delta^{2}H-a\delta^{18}O-b \end{array}$$

where δ^2^H and δ^18^O represent the sample isotope, while a and b denote the slope and intercept of the LMWL.

#### 2.3.3 Craig-Gordon model

Based on the principle of isotopic mass balance, the Craig-Gordon model is applied to quantitatively calculate the soil evaporation loss rate (Gonfiantini, 1986; Sprenger et al., 2017) (Equation 3):

(3) $$\begin{array}{c} f=1-{\left[\frac{\left(\delta_{s}-\delta^{*}\right)}{\left(\delta_{p}-\delta^{*}\right)}\right]}^{m} \end{array}$$

where δs represents the mean soil water isotopic across different soil depths, and δp corresponds to the original water source isotopic, determined by the intercept derived from the intersection point of the LMWL and Soil Evaporation Line (SEL) in an open liquid-vapor isotopic system (Evaristo et al., 2015; Javaux et al., 2016) (Equations 4, 5).

(4) $$\begin{array}{c} \delta^{18}O_{intercept}=\frac{d-b}{a-c} \end{array}$$

(5) $$\begin{array}{c} \delta^{2}H_{intercept}=a\delta^{18}O_{intercept}+b \end{array}$$

where c and d are the slope and intercept of the SEL, respectively, and all other parameters retain their previously defined meanings. The slope and intercept of the SEL can be calculated by the following formula (Gonfiantini, 1986) (Equations 6, 7):

(6) $$\begin{array}{c} c=\frac{{\left[\frac{h\left(\delta_{A}-\delta_{p}\right)-\left(1+\delta_{p}\times 10^{-3}\right)\left(\epsilon_{k}+\epsilon^{+}/\alpha^{+}\right)}{1-h+\epsilon_{k}\times 10^{-3}}\right]}_{2}}{{\left[\frac{h\left(\delta_{A}-\delta_{p}\right)-\left(1+\delta_{p}\times 10^{-3}\right)\left(\epsilon_{k}+\epsilon^{+}/\alpha^{+}\right)}{1-h+\epsilon_{k}\times 10^{-3}}\right]}_{18}} \end{array}$$

(7) $$\begin{array}{c} d=\delta^{2}H_{s}-m\times \delta^{18}O_{s} \end{array}$$

where h is the relative humidity of ambient air, and δA is the isotopic composition of atmospheric vapor, calculated as (Equation 8):

(8) $$\begin{array}{c} \delta_{A}=\frac{\delta_{rain}-k\epsilon^{+}}{1+k\alpha^{+}\times 10^{-3}} \end{array}$$

where δrain is the isotope values in precipitation, and ϵ+ and α+ are the equilibrium fractionation coefficients (Horita and Wesolowski, 1994) (Equations 9–11):

(9) $$\begin{array}{c} \epsilon^{+}=\left(\alpha^{+}-1\right)\times 1000 \end{array}$$

(10)  103ln[α+(H 2)]=1158.8(T3/109)−1620.1(T2/106)+794.84(T/103)−16104 + 2.9992(109/T3)

(11) 103ln[α+(O 18)]=−7.685+6.7123(103/T)−1.6664(106/T2)+0.3504(109/T3)

where T is the air temperature (K).

δ* is the limiting factor of isotope enrichment (Gonfiantini, 1986) (Equation 12):

(12) $$\begin{array}{c} \delta^{*}=\frac{h\delta_{A}+\epsilon_{k}+\epsilon^{+}/\alpha^{+}}{h-10^{-3}\left(\epsilon_{k}+\epsilon^{+}/\alpha^{+}\right)} \end{array}$$

ϵk is the kinetic fractionation coefficient (Merlivat, 1978) (Equations 13, 14):

(13) ϵk(H 2)=n(1−h)×(1−0.9755)×103(‰)

(14) ϵk(O 18)=n(1−h)×(1−0.9723)×103(‰)

The parameter n characterizes the aerodynamic conditions at the liquid-vapor interface during evaporation, while n is 1.0 for completely dry soils and 0.5 for lakes or saturated soils (Benettin et al., 2018), its value varies dynamically in natural settings due to alternating wetting-drying cycles driven by precipitation infiltration and soil evaporation. Consequently, this study adopted a mean n value of 0.75, consistent with field-based estimates (Mahindawansha et al., 2020).

m is the slope of enrichment (Gibson and Reid, 2014; Gibson et al., 2016) (Equation 15):

(15) $$\begin{array}{c} m=\frac{h-\left(\epsilon_{k}+\epsilon^{+}/\alpha^{+}\right)\times 10^{-3}}{1-h+\epsilon_{k}\times 10^{-3}} \end{array}$$

All parameters in the equation have been explained above.

#### 2.3.4 MixSIAR model

MixSIAR employs a Bayesian statistical framework that quantifies uncertainties in source contributions, isotopic signatures, and fractionation factors, and provides full posterior distributions to support statistical comparisons among groups. This approach is more robust than the feasible solution ranges provided by IsoSource. In addition, the model allows the integration of prior information to improve estimation accuracy, can simultaneously partition multiple water sources, and incorporates continuous covariates such as time and space. These features make MixSIAR well-suited for the complex scenarios examined in this study, which involve multiple water sources (precipitation, soil water at different depths, and groundwater) and comparisons across stand types and sampling months (Stock and Semmens, 2016). To ensure the robustness of these modeling outputs, careful consideration was given to the selection of the isotopic tracer. δ^18^O was chosen as the primary tracer for two main reasons. First, the strong linear covariation between δ²H and δ^18^O in xylem water indicated that either isotope could reliably represent plant water sources, making δ^18^O a suitable and equivalent choice. Second, despite applying spectral correction algorithms to address potential organic contamination in xylem water samples, δ^18^O measurements are inherently less susceptible to such analytical artifacts than δ²H, thereby providing a more robust dataset for source partitioning. The combination of strong isotopic covariation and superior analytical reliability justifies the use of δ^18^O as the primary tracer in this study. Fractionation factor was set to 0‰ based on the widely accepted premise that no isotopic fractionation occurs during water uptake by plant roots under non-saline conditions (Dawson and Ehleringer, 1993), Chen et al. (2020) demonstrated through controlled experiments that significant δ^18^O offset does not occur between plants and water sources. Markov chain Monte Carlo (MCMC) run length was set to “long”, and the model error structure was selected as “process + residual”. Convergence and stability of MCMC chains are critically assessed. The Geweke diagnostic compares early and late segments of a single chain using a z-score, with values beyond ±1.96 suggesting non-convergence. The Gelman-Rubin diagnostic evaluates between-chain and within-chain variances, with a potential scale reduction factor below 1.05 indicating convergence.

## 3 Results

### 3.1 Soil moisture content and isotopes

**Figure 2:**
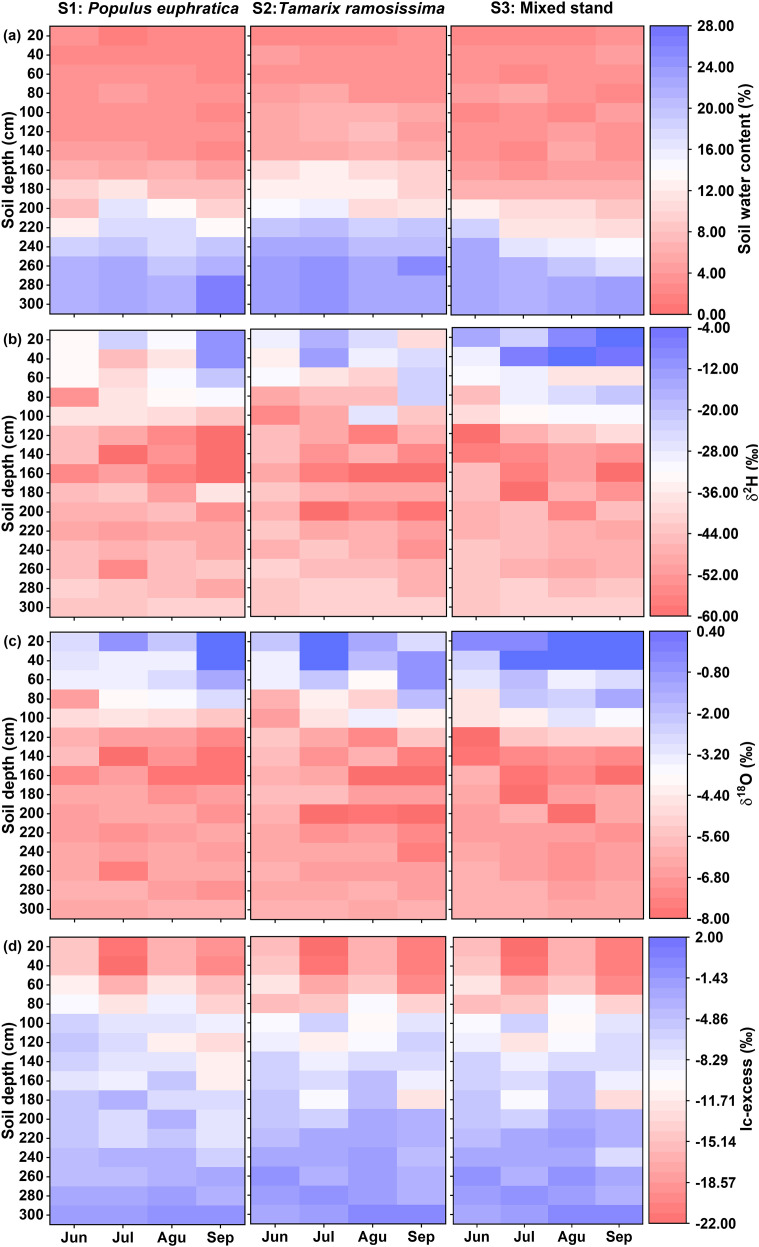
Vertical distribution of soil water content **(A)**, δ²H **(B)**, δ^18^O **(C)** and lc-excess **(D)**.

**Figure 3:**
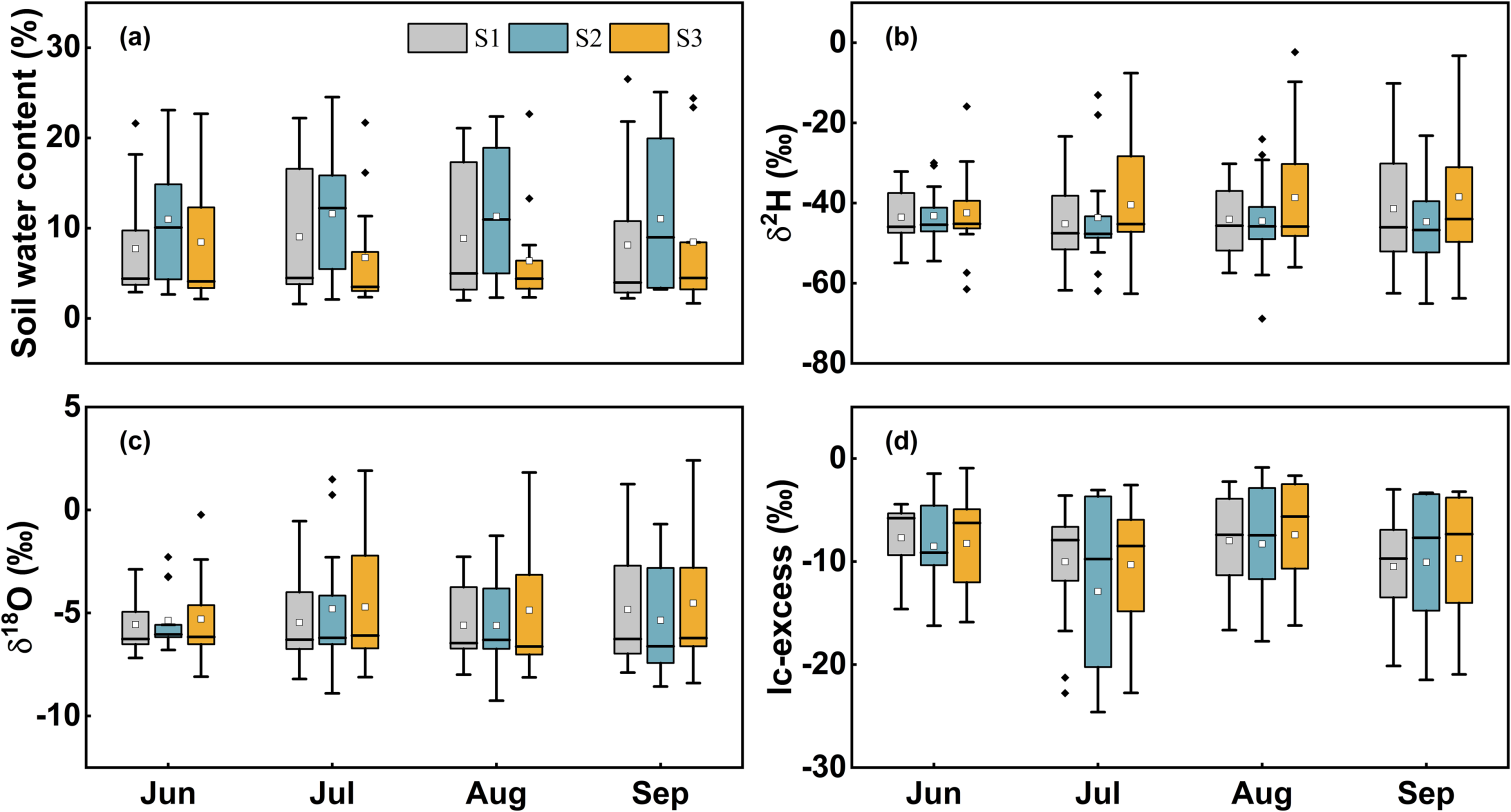
The variation of soil water content **(A)**, δ²H **(B)**, δ^18^O **(C)** and lc-excess **(D)** from June to September.

Soil water content (SWC) was monitored at three sites and exhibited highly consistent vertical profiles with minimal monthly variation (**Figures 2**, **3**). Influenced by shallow groundwater dynamics, all sites showed a distinct turning point depth (averaging 160 cm, 140 cm, and 180 cm, respectively), separating two hydrological zones. Above this depth, severe moisture stress was observed, with critically low mean SWC values (S1: 3.59%; S2: 4.49%; S3: 3.78%). Below it, SWC increased exponentially with depth (R² > 0.84, *p* < 0.01), indicating a shift from unsaturated to capillary-saturated conditions, which strongly influenced deep-rooted water uptake strategies. Vertical δ^18^O profiles revealed a stratified pattern, initially decreasing and then increasing with depth (**Figure 2**). The surface layer (0–40 cm) showed pronounced isotopic enrichment (S1: –3.48‰ to 1.25‰; S2: –3.22‰ to 1.48‰; S3: –2.51‰ to 2.40‰), resulting from intense evaporation. The subsurface layer (40–100 cm) exhibited the largest δ^18^O variability (S1: –6.72‰ to –1.19‰; S2: –6.79‰ to –0.69‰; S3: –4.63‰ to –1.30‰), suggesting mixing of heterogeneous water sources. The lowest δ^18^O values occurred at 100–200 cm (S1: –8.19‰ to –4.61‰; S2: –9.25‰ to –5.53‰; S3: –8.40‰ to –5.12‰), while values below 200 cm were more homogeneous (S1: –7.58‰ to –6.26‰; S2: –7.50‰ to –6.20‰; S3: –7.10‰ to –6.31‰), indicating stable hydraulic connectivity with groundwater. One-way ANOVA showed no statistically meaningful differences in δ^18^O among equivalent soil layers below 40 cm (*p* > 0.05), with marked heterogeneity confined to the surface horizon.

### 3.2 Hydrogen and oxygen isotope relationships

**Figure 4:**
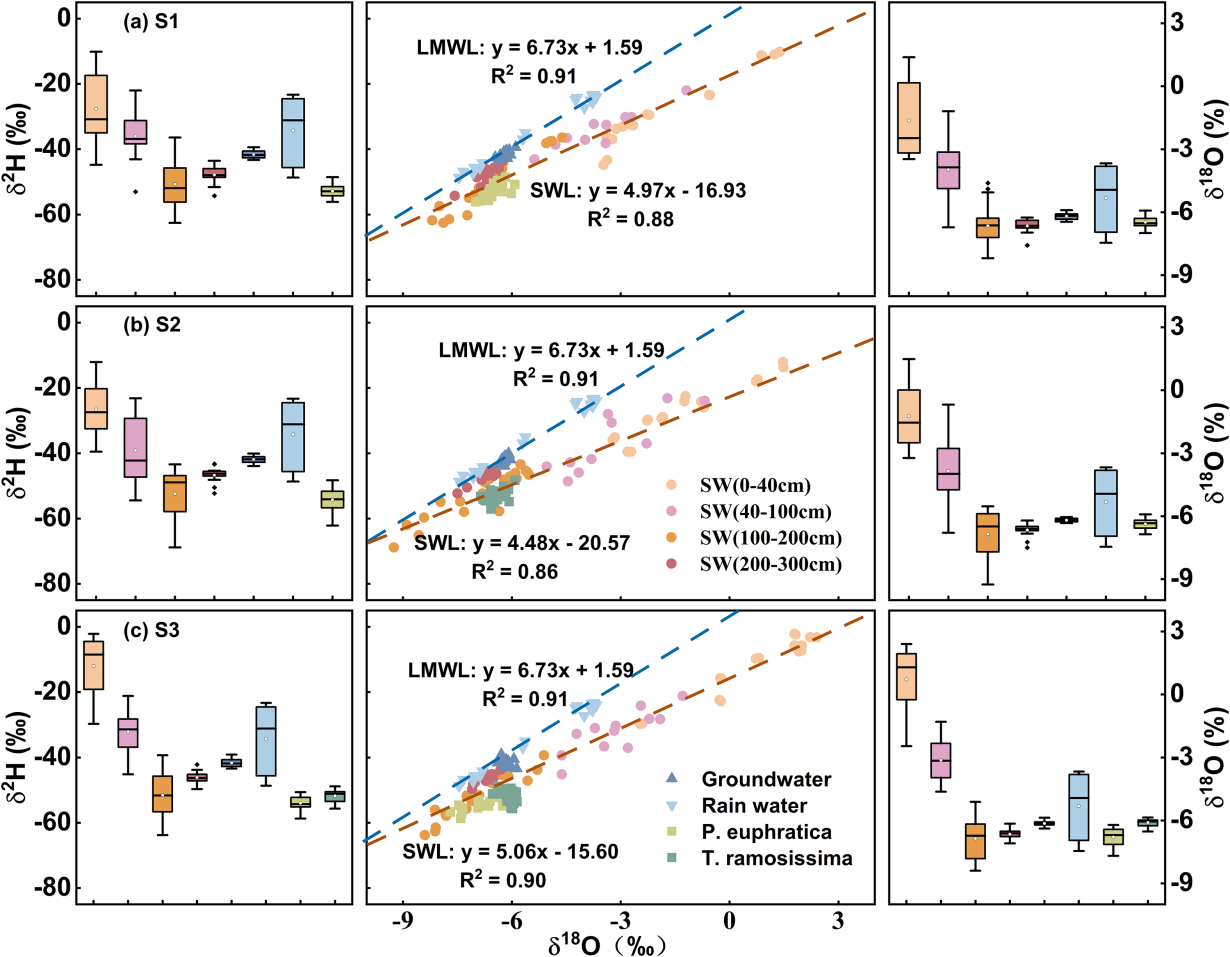
The relationship between δ^2^H and δ^18^O in S1: Pure *P. euphratica* **(A)**, S2: Pure *T. ramosissima* **(B)** and S3: mixed forest **(C)**. Note: LMWL is local meteoric water line and SWL is soil water line.

To clarify the environmental significance of stable isotope in precipitation, soil water, groundwater, and xylem water, and to assess hydrological connectivity among these compartments, this study established a Local Meteoric Water Line (LMWL) and Soil Water Lines (SWL) based on δ²H–δ^18^O covariation analysis (**Figure 4**, **Table 2**). Precipitation samples collected during the observation period yielded the LMWL: δ²H = 6.73δ^18^O + 1.59, with δ^18^O values ranging from –7.45‰ to –3.67‰ and δ²H from –48.66‰ to –23.30‰. Compared to the Global Meteoric Water Line (GMWL: δ²H = 8δ^18^O + 10), the LMWL showed a lower slope (6.73) and intercept (1.59), which was indicative of substantial sub-cloud evaporation during rainfall events, a signature of continental moisture deficit typical of arid climates. The SWL exhibited marked spatial heterogeneity across the study area, with slopes of 4.97 (S1), 4.48 (S2), and 5.06 (S3), all significantly lower than that of the LMWL (*p* < 0.05, **Figure 4**), reflected progressive isotopic enrichment due to soil evaporation. Notably, the *T. ramosissima* stand (S2) had the shallowest SWL slope (4.48), indicating the strongest evaporation effect. One-way ANOVA confirmed distinct monthly variation in SWL slopes (*p* < 0.05), with the minimum slope occurring in July, consistent with enhanced evaporative fractionation under high summer temperatures, illustrating a thermal-seasonal control mechanism on soil water isotopes. Groundwater isotopes plotted to the right of the LMWL (**Figure 4**), with narrow value ranges across sites (S1: –6.34‰ to –5.90‰; S2: –6.33‰ to –6.04‰; S3: –6.38‰ to –5.87‰), indicating high hydrological stability in the aquifer. Given strong δ²H–δ^18^O covariation in xylem water, δ^18^O was used as the primary tracer. Xylem water analysis showed that *P. euphratica* in pure stand (S2: –6.99‰ to –5.92‰) was slightly enriched compared to mixed stand (S3: –7.69‰ to –6.22‰). In contrast, *T. ramosissima* in mixed forest (S2: –6.86‰ to –5.92‰) was more depleted than in mixed stand (S3: –6.52‰ to –5.86‰). Significant overlaps were found between xylem and isotopes in 100–300 cm soil water and groundwater (*p* < 0.01), confirming that these woody phreatophytes primarily rely on deep subsurface water for transpiration.

**Table 2:** Soil water lines (SWL) in *P. euphratica* (S1), *T. ramosissima* (S2), and mixed (S3) stands from June to September.

Site	June	July	August	September
S1	y = 4.58x – 17.38 R^2^ = 0.85	y = 4.06x – 21.98 R^2^ = 0.82	y = 4.52x – 17.58 R^2^ = 0.89	y = 5.44x – 15.13 R^2^ = 0.93
S2	y = 4.23x – 19.74 R^2^ = 0.81	y = 4.08x – 22.63 R^2^ = 0.84	y = 4.95x – 17.23 R^2^ = 0.87	y = 4.32x – 20.55 R^2^ = 0.91
S3	y = 4.91x – 15.63 R^2^ = 0.94	y = 4.73x –17.30 R^2^ = 0.87	y = 5.04x – 13.23 R^2^ = 0.88	y = 4.95x – 15.35 R^2^ = 0.87

### 3.3 Soil evaporation loss rates and influence factors

**Figure 5:**
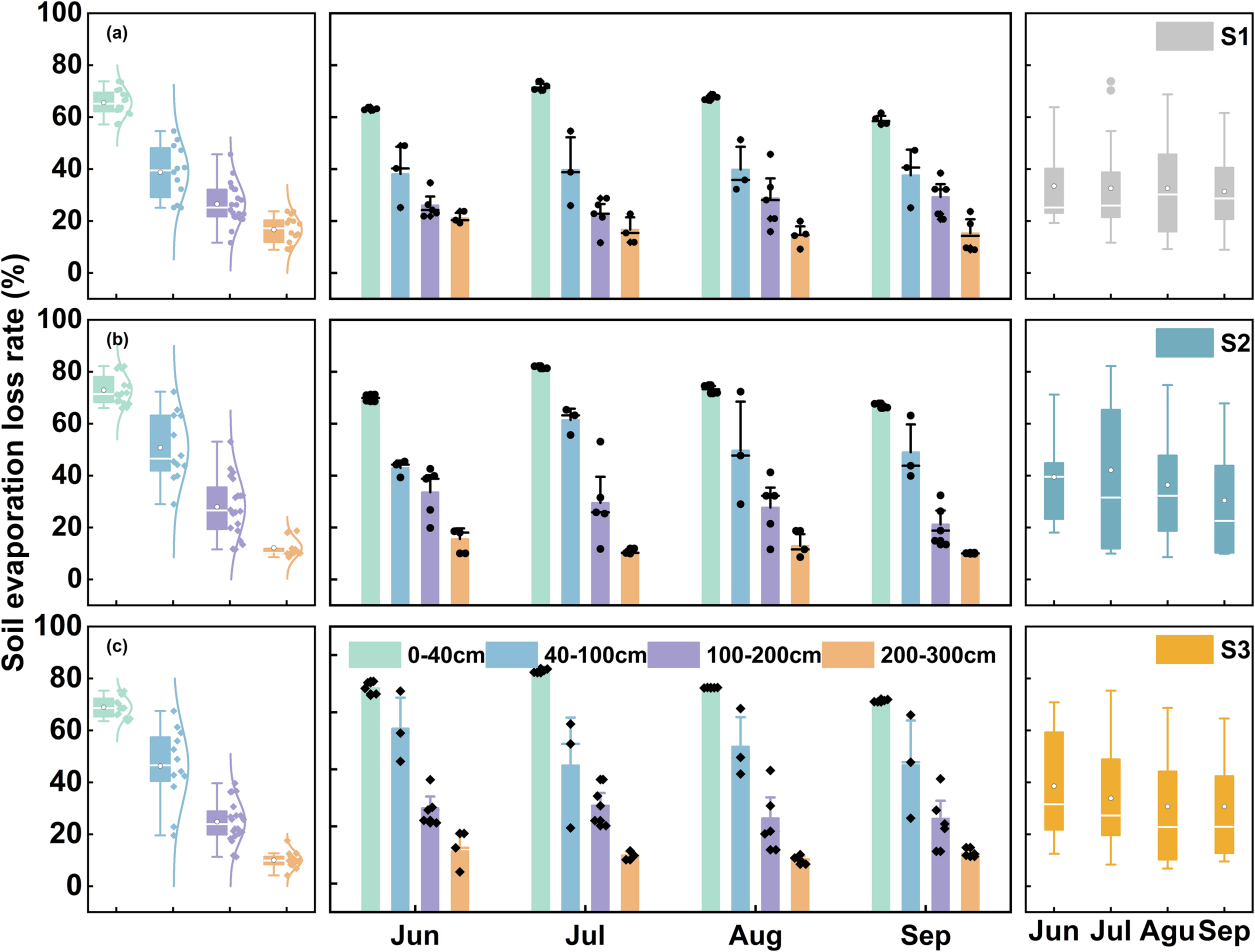
Monthly variation of evaporation loss rates in various layers (middle), depth variation (left) and monthly variation (right) at S1: Pure *P. euphratica* **(A)**, S2: Pure *T. ramosissima* **(B)** and S3: mixed forest **(C)**.

This study, through an analysis of the vertical differentiation patterns of soil water lc-excess and evaporative loss rates (f), revealed that both parameters exhibit an exponential attenuation of soil evaporative fractionation effects with increasing depth (**Figure 2**). Measured data confirmed pervasive strong evaporation across the region, as evidenced by consistently negative lc-excess values ranging from –24.62‰ to –1.67‰ in all sampled soil layers (**Figure 2**). Within the 0–40 cm topsoil layer, direct atmospheric exposure induced intense evaporative perturbation, resulting in substantial isotopic enrichment (mean lc-excess: –18.08‰, –19.83‰, –18.70‰). Driven by seasonal variations in solar radiation and evaporative demand, f showed considerable temporal variability, displaying a unimodal distribution peaking at 76.12% ± 4.27% in July and reaching a minimum of 63.46% ± 3.42% in September. With increasing depth into the 40–100 cm layer, lc-excess demonstrated progressive enrichment with diminishing increments, rising to site means of –10.79‰, –14.84‰, and –12.63‰. This was concurrent with substantial reductions in f relative to surface values, with decreases of 26.75%, 22.14%, and 22.61% observed across the three sites. Progressing to the 100–200 cm layer, lc-excess continued its ascent to –7.50‰, –7.76‰, and –7.01‰, while f values declined synchronously to 26.53%, 27.98%, and 24.85%, accompanied by attenuated seasonal fluctuations. At depths exceeding 200 cm, lc-excess stabilized, showing minimal deviation from local groundwater, and the evaporative process became essentially stagnant, yielding mean f values of only 14.61%, 11.79%, and 10.52% for the three sites. This implied hydraulic connectivity and suggested that isotopic composition in this zone was predominantly governed by groundwater recharge processes (**Figure 5**). Vegetation exerted limited influence, within the 0–100 cm layer, the *T. ramosissima* stand (S2) exhibited a marginally higher evaporative loss rate compared to other sites, although the statistical difference was not statistically significant (*p* > 0.05). Below 200 cm, the *P. euphratica* forest (S1) showed a slightly higher f value than other sites (*p* > 0.05), while f at all sites remained below 15% (**Figure 5**). Collectively, these findings indicated spatially homogeneous hydrogeological conditions across the study area, where vertical hydrological fluxes predominated over local ecological factors in governing deep soil water movement.

**Figure 6:**
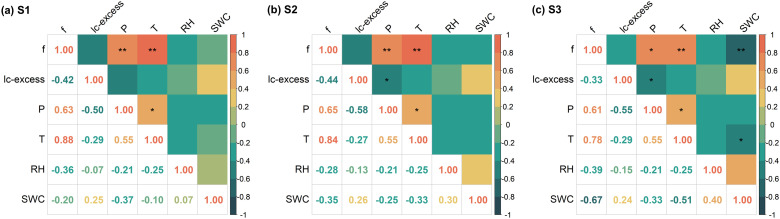
The correlation between soil evaporation loss rates and environmental factors S1: Pure *P. euphratica* **(A)**, S2: Pure *T. ramosissima* **(B)** and S3: mixed forest **(C)**. Note: f is evaporative loss rates, lc-excess is line-conditioned excess, P is precipitation, T is temperature, RH is relative humidity and SWC is soil water content.

The evaporative fractionation process in surface soil layers was considerably more complex than in deeper layers (Sprenger et al., 2017), owing primarily to direct exposure to dynamic meteorological conditions, which heightened environmental sensitivity. Accordingly, this study employed correlation analysis to elucidate the regulatory mechanisms of environmental factors controlling f in the 0–40 cm surface layer of an extremely arid region (**Figure 6**). The results indicated that air temperature and precipitation were the principal climatic drivers of evaporative fractionation in surface soils. Statistically strong positive correlations were observed between f and mean air temperature across all three sites (*p* < 0.05), along with similarly strong positive correlations with precipitation (*p* < 0.05). This coupling was most pronounced in July, coinciding with seasonal peaks in both temperature and precipitation. During this period, elevated temperatures accelerated soil water phase changes, while precipitation replenished surface moisture, both contributing to higher f values compared to other months. Relative humidity showed weakly negative correlations with f (r = –0.35, –0.27, –0.41), suggesting that high atmospheric humidity may suppress evaporation by reducing the vapor pressure gradient. However, the lack of statistical significance precluded its identification as a major regulatory factor. Although a negative feedback relationship between soil water content (SWC) and f was theoretically expected, the persistently hyper-arid conditions led to chronically low SWC levels in surface soils. This hydrological stability effectively masked the functional influence of SWC on evaporation dynamics, resulting in non-significant correlations (*p* > 0.05) at all sites except S3, where a moderate negative correlation was marked (r = –0.58, *p* < 0.05).

### 3.4 Comparison of water use patterns in pure and multi-species stands

**Figure 7:**
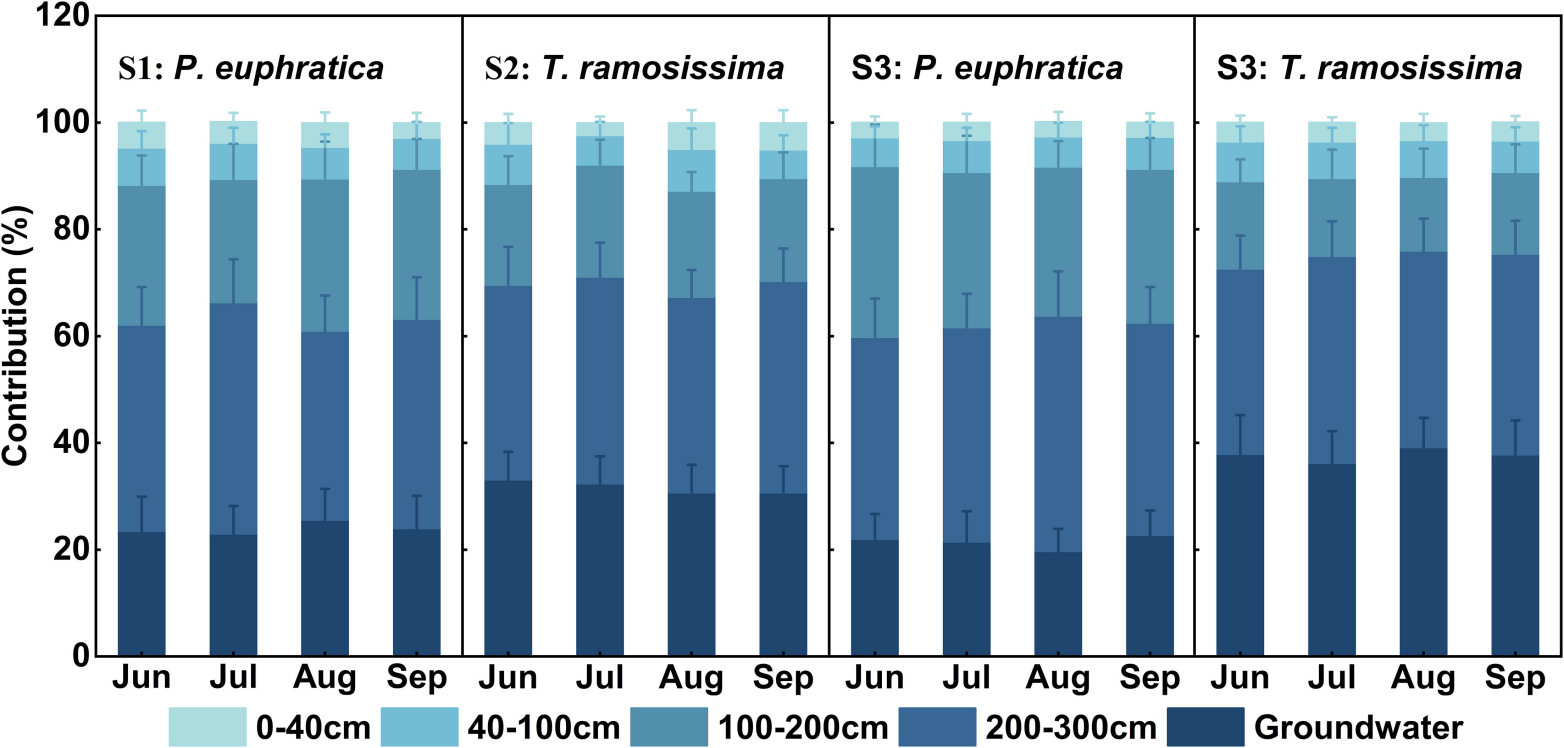
Monthly variation in soil water and groundwater utilization by *P. euphratica* and *T. ramosissima* in pure and mixed stands.

**Figure 8:**
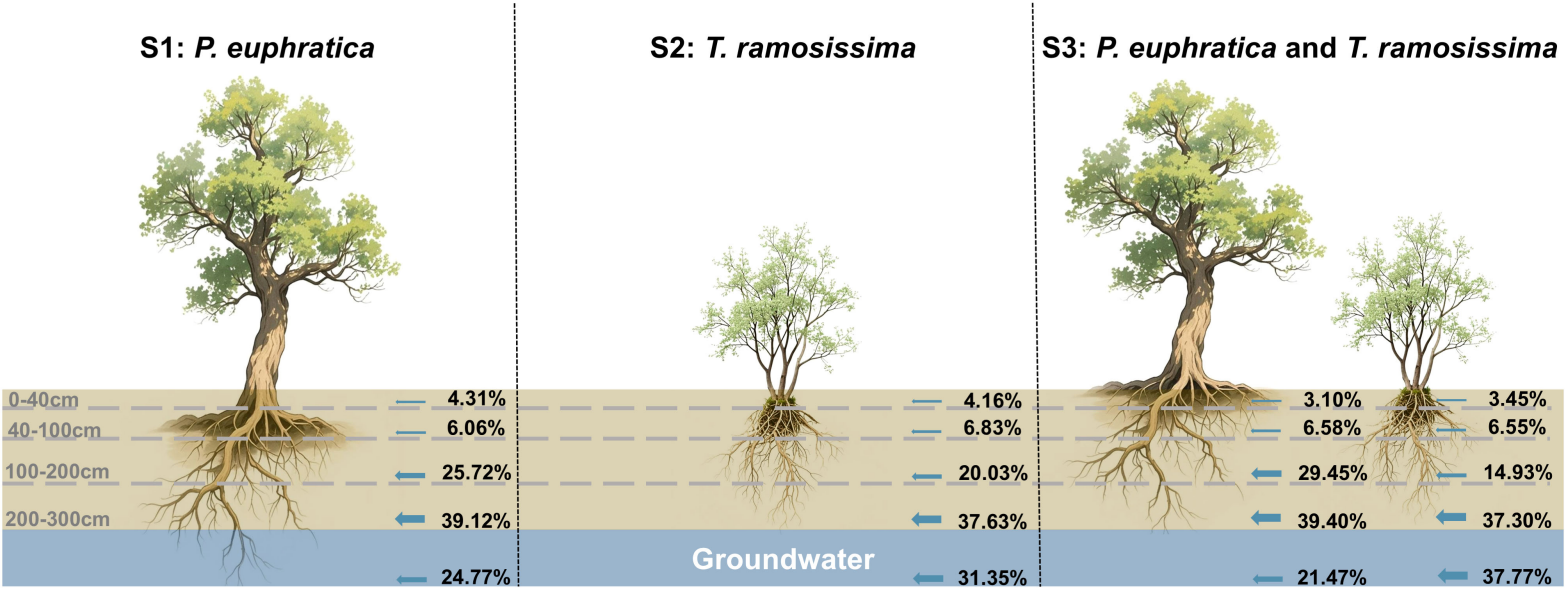
Potential water source contribution of *P. euphratica* and *T. ramosissima* at different stands.

Quantitative analysis using the MixSIAR model revealed distinct water-use strategies between *P. euphratica* and *T. ramosissima* across pure and mixed stands (**Figures 7**, **8**), with the principal differentiation manifested in their proportional uptake of intermediate-depth soil water (100–200 cm) and groundwater. In pure stands, both species exhibited a conservative water acquisition pattern characterized by predominant reliance on deep soil water (200–300 cm) and groundwater, though interspecific variation was already evident. For *P. euphratica* (S1), deep soil water contributed the largest proportion (39.12%), followed by intermediate soil water (25.72%) and groundwater (24.77%). For *T. ramosissima* (S2), deep soil water also dominated (37.63%), but groundwater contributed considerably more (31.35%) than for *P. euphratica*, while intermediate soil water contributed less (20.03%). In mixed stands (S3), water-use strategies of both species underwent marked and antagonistic shifts (*p* < 0.05), amplifying the divergence observed in pure stands. Specifically, *P. euphratica* increased its reliance on intermediate soil water (from 25.72% to 29.45%) while reducing groundwater use (from 24.77% to 21.47%). Conversely, *T. ramosissima* increased groundwater uptake (from 31.35% to 37.77%) and decreased intermediate soil water use (from 20.03% to 14.93%). Direct comparison of the two species in mixed stands further highlighted pronounced differences in water-source partitioning, *P. euphratica* derived nearly double the proportion of its water from the intermediate soil layer compared to *T. ramosissima* (29.45% and 14.93%, respectively), whereas *T. ramosissima* relied more heavily on groundwater (37.77% and 21.47%, respectively). In contrast, both species exhibited comparable contributions from deep soil water (39.40% compared to and 37.30%) and shallow soil water (0–100 cm), indicating that niche differentiation was driven primarily by shifts in intermediate soil water and groundwater utilization. Throughout the study period, both species maintained consistent seasonal water-use dynamics, a pattern potentially attributable to relatively stable soil moisture profiles and groundwater levels during the observational timeframe.

## 4 Discussion

### 4.1 Soil evaporation under different vegetation conditions

In arid and semi-arid regions, water scarcity leads to soil evaporation becoming a dominant process governing vertical moisture transport and the spatiotemporal distribution of regional water resources (Chang et al., 2019; Dawson and Pate, 1996; Tugwell-Wootton et al., 2020). Soil water isotopes (δ²H and δ^18^O) identify water movement mechanisms like infiltration and evaporation across depths (Ala-Aho et al., 2018; Al-Oqaili et al., 2020). Across all vegetation types within the study area, SWL slopes were significantly lower than LMWL (*p* < 0.05, **Figure 4**), indicating widespread evaporative fractionation (Wang et al., 2017; Zhu et al., 2021). The SWL slope indirectly indicates evaporation intensity, with lower values mean stronger non-equilibrium evaporation and less residual moisture (Gibson et al., 2008). Evaporation intensity across the three sites ranked as follows: the *T. ramosissima* stand (S2: 4.48) > the *P. euphratica* stand (S1: 4.98) > the mixed forest (S3: 5.06), showing vegetation type modulates evaporation by micro-environmental conditions (Geris et al., 2017; Huang et al., 2023). The line-conditioned excess (lc-excess) within soil profiles under different vegetation types reflected evaporation processes driven by drought during the study period. Despite differences in vegetation cover, lc-excess values showed similar vertical patterns: high variability in the upper 40 cm, decreasing markedly with depth, and stabilizing below 2 m (**Figure 2**). The 0–40 cm layer consistently had the lowest average lc-excess, identifying it as the most intense evaporation zone. Analysis of evaporation loss rates (f) revealed that the *T. ramosissima* stand (S2) had the highest f (**Figure 5**), consistent with its canopy structure.

The peak f observed in July coincided with fluctuations in precipitation, air temperature, and humidity (Benettin et al., 2018; Geris et al., 2017; Wu et al., 2016). Correlation analysis (**Figure 6**) showed a strong positive relationship between f in the 0–40 cm layer and precipitation, which is typical in arid regions where shallow infiltration from short rain events combines with high radiation and temperatures to drive rapid surface evaporation. These findings are consistent with those of Qiu et al. (2023), who investigated soil evaporation dynamics in an artificial forest within the arid oasis ecosystem of Shiyang River Basin, Northwest China. Their study similarly reported that elevated temperatures intensified evaporative enrichment of soil water isotopes. This thermodynamic effect, whereby higher air temperatures enhance vapor diffusion, saturated vapor density, and hydraulic conductivity, collectively accelerating evaporation and increasing the evaporation-to-transpiration ratio (f) (Berkelhammer et al., 2016), has been widely documented across arid regions (Canet-Martí et al., 2023; Li et al., 2025). Comparable evaporative patterns have also been observed in different vegetation zones in the Qilian Mountains (Zhu et al., 2022) and the Tengger Desert, northwestern China (Pan et al., 2023), further supporting the generalizability of these mechanisms. Although increased relative humidity suppressed f by reducing vapor diffusion (Bao et al., 2019), this effect was weak and often overshadowed by the dominant factors of high radiation, temperature, and limited soil moisture in this region. Theoretically, surface soil water content (SWC) should control evaporation, but persistent surface desiccation meant evaporation was limited by vapor diffusion, not liquid supply (Zhang et al., 2024). Thus, evaporation was insensitive to minor SWC variations, with soil properties and vegetation cover weakening the expected correlation (Zhou et al., 2023). Evaporation rates decreased exponentially with depth, having negligible influence on deeper layers (**Figure 2**). In deeper layer, moisture moved upward mainly as adsorbed films or vapor, with minimal capillary rise (Du et al., 2018). Once moisture passed through the dry surface layer, it was lost to the atmosphere through slow molecular diffusion, resulting in very low evaporation fluxes. Deep soil layers near the groundwater table showed lc-excess values near zero and f values generally below 12% (**Figure 2**, **5**), implying that evaporation signals were obscured by upward recharge from non-evaporative groundwater (Dai, 2011; Huang et al., 2023; Martínez-de la Torre and Miguez-Macho, 2019). Consequently, deep-soil isotope estimates may underestimate true evaporation fractions.

### 4.2 Water use patterns in *P. euphratica* and *T. ramosissima* stands

Plant water use characteristics were influenced by soil water content, groundwater table, seasonal rainfall variations, and root distribution (Pei et al., 2023). During the study period, the groundwater table remained stable at approximately 3 m (**Figure 2**), making both soil water and groundwater accessible for the vegetation. Under these relatively stable moisture conditions, *P. euphratica* and *T. ramosissima* exhibited consistent proportional water source utilization across months in both pure and mixed stands (**Figure 7**, **8**). Although the xylem δ^18^O values of both species did not differ significantly (*p* > 0.05, **Figure 4**), suggesting similar water-use strategies with primary extraction from the 100–300 cm depth, their specific water source preferences differed. *P. euphratica* derived more water from soil at 100–200 cm, while *T. ramosissima* relied more heavily on groundwater, indicating that *P. euphratica* preferentially utilizes soil water and *T. ramosissima* specializes in accessing deeper, more stable sources. In the mixed stand, this interspecific divergence was more pronounced. Compared to pure stands, *P. euphratica* shifted uptake toward to the 100–200 cm layer and reduced groundwater dependence, while *T. ramosissima* increased groundwater use and decreased reliance on the 100–200 cm soil water. These contrasting responses can be explained by differences in hydraulic architecture and root system development between the two species. In terms of hydraulic architecture, *P. euphratica* exhibits relatively high xylem hydraulic conductivity in shallow to intermediate roots, facilitating efficient extraction of soil water from upper layers (Ayup et al., 2015; Li et al., 2019b). However, this species is more vulnerable to xylem embolism under severe water stress, which may constrain its ability to rely exclusively on deeper, more stable water sources when competition intensifies (Rajput et al., 2015; Zhou et al., 2013). In contrast, *T. ramosissima* possesses greater embolism resistance and a more conservative xylem structure, allowing sustained water transport under low water potentials (Ayup et al., 2015; Rzepecki et al., 2011). This species also exhibits higher hydraulic safety margins, enabling it to maintain hydraulic conductivity even when groundwater levels decline or soil moisture becomes limiting (Akhmedov et al., 2025; Yu et al., 2013). These hydraulic contrasts are complemented by differences in root architecture (Chen et al., 2016; Wang et al., 2021). *P. euphratica* develops extensive lateral roots extending 30–50 m, maximizing spatial acquisition of soil moisture from upper and intermediate layers (Ye et al., 2019; Hukin et al., 2005). *T. ramosissima* exhibits strong root hydrotropism, concentrating roots in layers with highest water availability (Dong et al., 2024). Under drought and competition, it prioritizes deep soil exploration, with roots penetrating beyond 10 m and a high root-to-shoot ratio ensuring stable access to deep water (Yuan et al., 2015; Liu et al., 2022).

The contrasting groundwater depth (GWD) adaptation thresholds between the two species substantiate their divergent ecohydrological strategies. *P. euphratica* has a narrow optimal growth zone at 3–4 m GWD, while *T. ramosissima* maintains robust growth from shallow aquifers to depths exceeding 6 m (Chen et al., 2016; Li et al., 2013; Zhu et al., 2009). These thresholds reflect underlying differences in rooting depth and hydraulic traits, the embolism resistance of *T. ramosissima* allows it to exploit deeper water sources that are either inaccessible or hydraulically risky for *P. euphratica* (Zhou et al., 2017). The observed niche differentiation, specifically the shift of *P. euphratica* toward intermediate soil water and *T. ramosissima* toward groundwater in mixed stands, represents a complementary resource use that may buffer against competitive exclusion in the short term (Guo et al., 2019; Wan et al., 2022). However, its effectiveness depends on environmental context. Under stable groundwater conditions, such partitioning could promote long-term coexistence by reducing direct competition for the same water sources (Chen et al., 2016; Tan et al., 2017). Beyond hydraulic traits and root distribution, other environmental drivers may also influence water-use strategies. Soil salinity, which varies spatially in the study area, can affect water uptake by altering osmotic potential and root permeability (Fu et al., 2012; Zhao et al., 2017). *T. ramosissima* is known for its higher salt tolerance, which may further enhance its competitive advantage in saline microsites (Cao et al., 2020; Shuyskaya et al., 2017). Microtopography and localized variations in soil texture can also create heterogeneity in water availability (Li et al., 2024; Zhang et al., 2023), potentially modulating the intensity of competition and the expression of niche differentiation (Li et al., 2019a). Under scenarios of continued groundwater drawdown or increased drought frequency, the competitive balance will likely shift further toward *T. ramosissima*, potentially triggering a regime shift in community composition (Wang et al., 2018). This aligns with broader ecological theory suggesting that environmental stress can modulate the outcome of niche partitioning, with harsher conditions favoring stress-tolerant species (Olsen et al., 2016; Wandrag et al., 2019). Such shifts would have profound consequences for ecosystem function, dominance by *T. ramosissima* may alter water cycling patterns, reduce habitat heterogeneity (Liu et al., 2024; Sun et al., 2022). Moreover, changes in species composition can affect belowground processes, including nutrient cycling and soil organic matter dynamics, with feedbacks to ecosystem productivity and stability (Li et al., 2024).

### 4.3 Inspiration for water resources management of desert riparian forest

Comprehensive meta-analyses demonstrate that increasing aridity alters interspecific interactions, leading to shifts in plant community dominance (Garssen et al., 2014). This trend is evident in the study area, located in the arid region of Northwest China, where chronic water deficit prevails. Precipitation is sparse, evaporation exceeds rainfall, and frequent extreme droughts intensify water scarcity, collectively restricting forest growth (Castillo et al., 2016; Han et al., 2021; Bravo-Avila and Feeley, 2023). The current dominance of mature riparian forests by *P. euphratica* is attributed to historical flooding regimes that facilitated its establishment (Li et al., 2013; Wu et al., 2016), with a groundwater depth of 2–4 m critical for its stable coexistence with *T. ramosissima* (Li et al., 2013). However, under persistent groundwater decline, *T. ramosissima* gains a competitive advantage through superior hydraulic efficiency and drought tolerance (Ayup et al., 2015). This competitive shift is influenced by current water management. Eco-water transfer projects have partially mitigated oasis degradation, current hydrological conditions remain insufficient to support the safe sites required for the sexual reproduction of *P. euphratica* (Liu et al., 2015; Wang et al., 2023). Anthropogenic water deliveries deviate significantly from natural flood regimes in timing and spatial distribution, often failing to align with periods of peak vegetation water demand. This discrepancy, combined with high surface soil evaporation, not only exacerbates water stress but also promotes soil salinization in oasis ecosystems (Yu et al., 2021). These conditions favor salt-tolerant *T. ramosissima* while impairing *P. euphratica* regeneration, driving its decline and enabling *T. ramosissima* expansion beyond its historical range (Lei et al., 2020). Consistent with this pattern, the present study confirms that *T. ramosissima* exhibits greater groundwater use efficiency and drought tolerance than *P. euphratica*. To alleviate the degradation of *P. euphratica* forests and maintain the stability of riparian ecosystems, current management strategies should prioritize optimizing ecological water allocation. This requires ensuring water availability during critical recruitment and early seedling establishment phases of *P. euphratica* through precise regulation of irrigation timing, volume, and frequency to promote suitable microhabitat conditions for population regeneration. Simultaneously, it is essential to maintain regional groundwater levels within species-specific ecological thresholds to support tree survival and long-term persistence of *P. euphratica*.

However, this study offered only a preliminary examination of evaporation processes and plant water sources in a desert riparian forest ecosystem dominated by *P. euphratica* and *T. ramosissima*, relying solely on stable hydrogen and oxygen isotope techniques. Nevertheless, several limitations should be considered when interpreting these findings. First, the MixSIAR model, while widely used for source partitioning, carries inherent uncertainties related to prior distributions, fractionation factors, and isotopic source variability. These factors may affect the precision of proportional contribution estimates, particularly when multiple water sources exhibit similar isotopic signatures. Second, our sampling was conducted only at plot scales over growing season, limiting our ability to capture interannual variability and spatial heterogeneity across the broader Heihe River Basin. Additionally, the relatively stable groundwater conditions during the study period constrain our assessment of plant water use responses under more extreme hydrological regimes. Third, this study focused exclusively on two keystone species (*P. euphratica* and *T. ramosissima*), leaving the water use strategies of other coexisting species and their interspecific interactions unexplored. Finally, the absence of direct physiological measurements, such as xylem hydraulic conductivity and *in situ* water potentials, limits mechanistic insights into species-specific drought responses. Future research should address these limitations through long term monitoring across multiple years and sites to capture interannual variability and assess plant water use plasticity under varying groundwater depths and climate conditions. Furthermore, integrating stable isotope approaches with direct physiological measurements, including hydraulic conductivity and stomatal conductance, would provide a more mechanistic understanding of plant water use responses to water stress.

## 5 Conclusion

This study elucidated the patterns of soil evaporation and plant water-use strategies in a desert riparian ecosystem dominated by *P. euphratica* and *T. ramosissima* using stable isotopes. The results demonstrate that evaporative losses are largely confined to shallow soil layers (0–40 cm) and are primarily controlled by regional hydrogeological conditions rather than local vegetation types. In deeper layers, groundwater recharge dominates, with negligible evaporation. Both species predominantly rely on water from the 100–300 cm soil layer and groundwater, but exhibit distinct differentiation, *P. euphratica* preferentially extracts soil water through its extensive lateral roots, while *T. ramosissima* shows greater dependence on groundwater through specialized vertical roots. This differentiation intensifies in mixed stands, leading to increased interspecific competition, reduced soil moisture, and faster groundwater decline, which may contribute to the ongoing degradation of *P. euphratica* populations and the expansion of *T. ramosissima*. These findings highlight that despite ecological water diversion projects mitigating overall ecosystem degradation, maintaining suitable groundwater levels is critical for sustaining *P. euphratica* dominance. Effective management should therefore optimize water conveyance to balance interspecific competition, thereby supporting the long-term stability of these fragile riparian forests.

## Data Availability

The raw data supporting the conclusions of this article will be made available by the authors, without undue reservation.
